# Supplementary material for: Effect of FKBP12-Derived Intracellular Peptides on Rapamycin-Induced FKBP–FRB Interaction and Autophagy
Source: Cells. 2022 Jan 24;11(3):385. doi: 10.3390/cells11030385 (PMC8834644; doi:10.3390/cells11030385)
Supplement: Supplementary file 1 [file cells-11-00385-s001.zip › cells-1494449-supplementary.pdf]

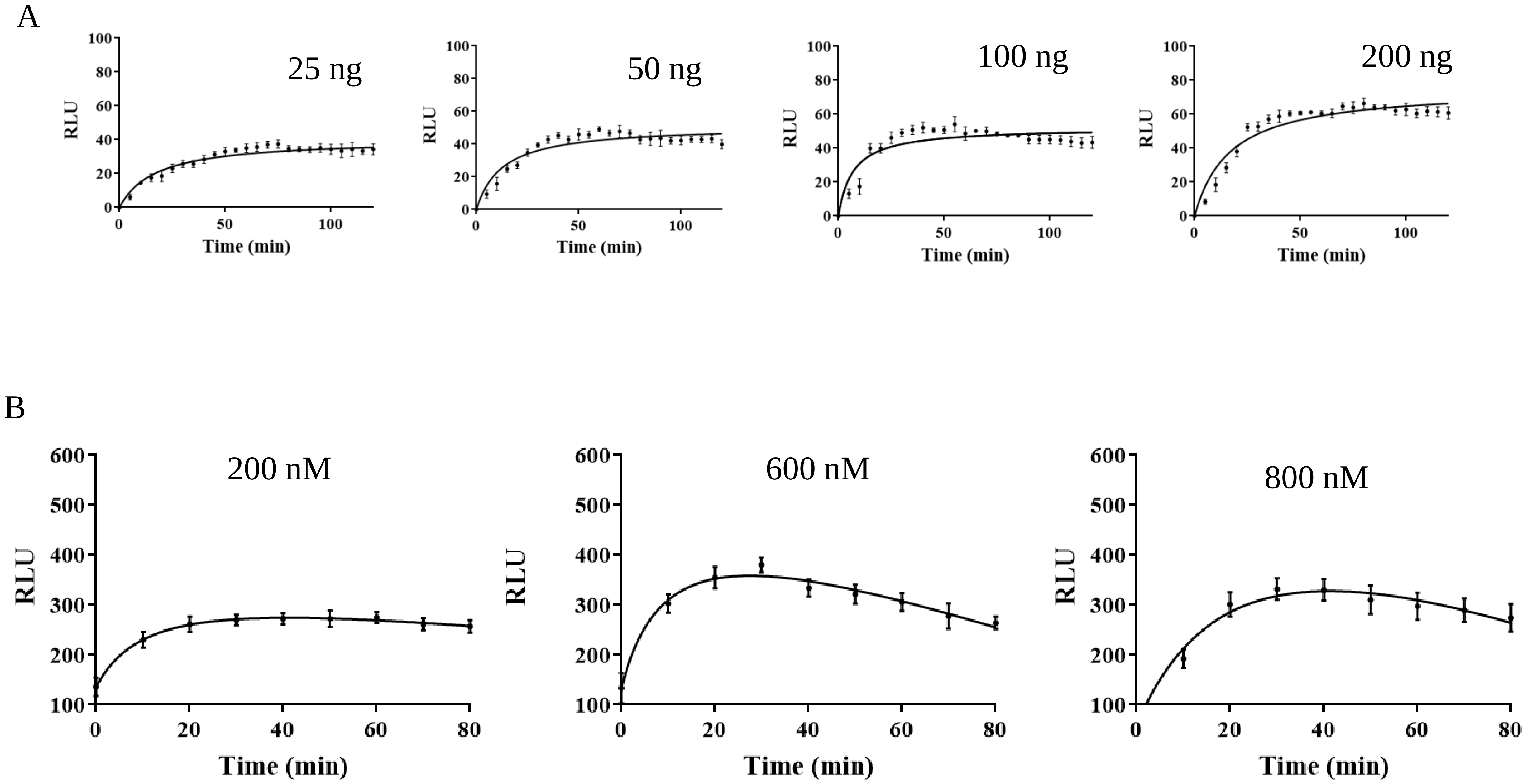

Supplemental Figure S1. Graphics shown the relative luminescent units (RLU) obtained with different concentrations of plasmids pair (A) or rapamycin (B). Additional details were shown on Experimental Procedures.

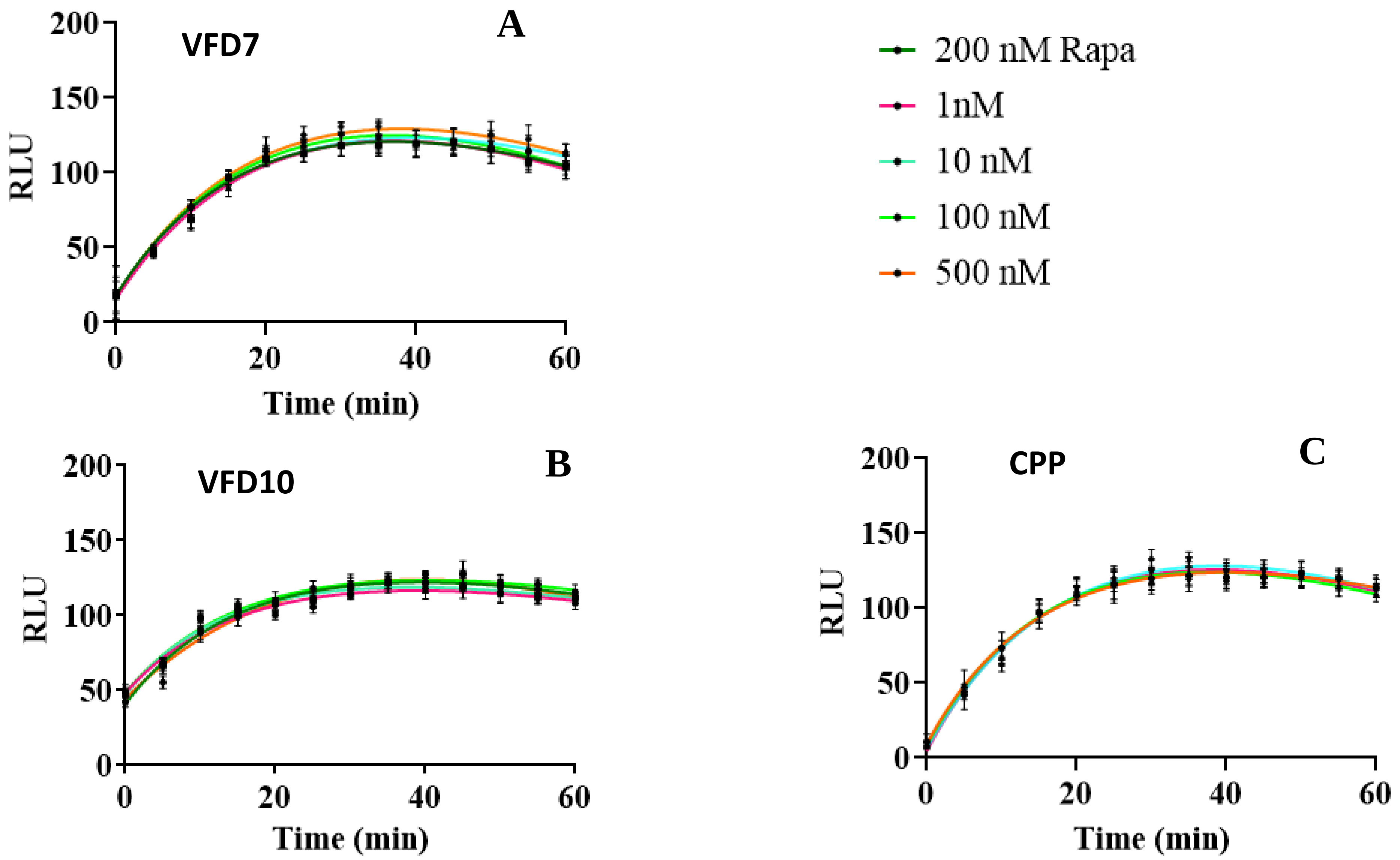

Supplemental Figure S2. Graphics shown the relative luminescent units (RLU) obtained with the indicated concentrations of cpp (a), VFD7 (b) or VFD10 (c). Note that none of the peptides were capable to alter the RLU compared to control rapamycin (Rapa) alone (a-c). Both VFD7 and VFD10 without the cpp covalently bound were also unable to inhibit the RLU induced by Rapa. Peptides VFA7, VFE7, VFD6, VFD5, VFD4, EVE5, or AVE5 have similar lack of activity without the presence of cpp covalently bound (data not shown). Additional details were shown on Experimental Procedures.

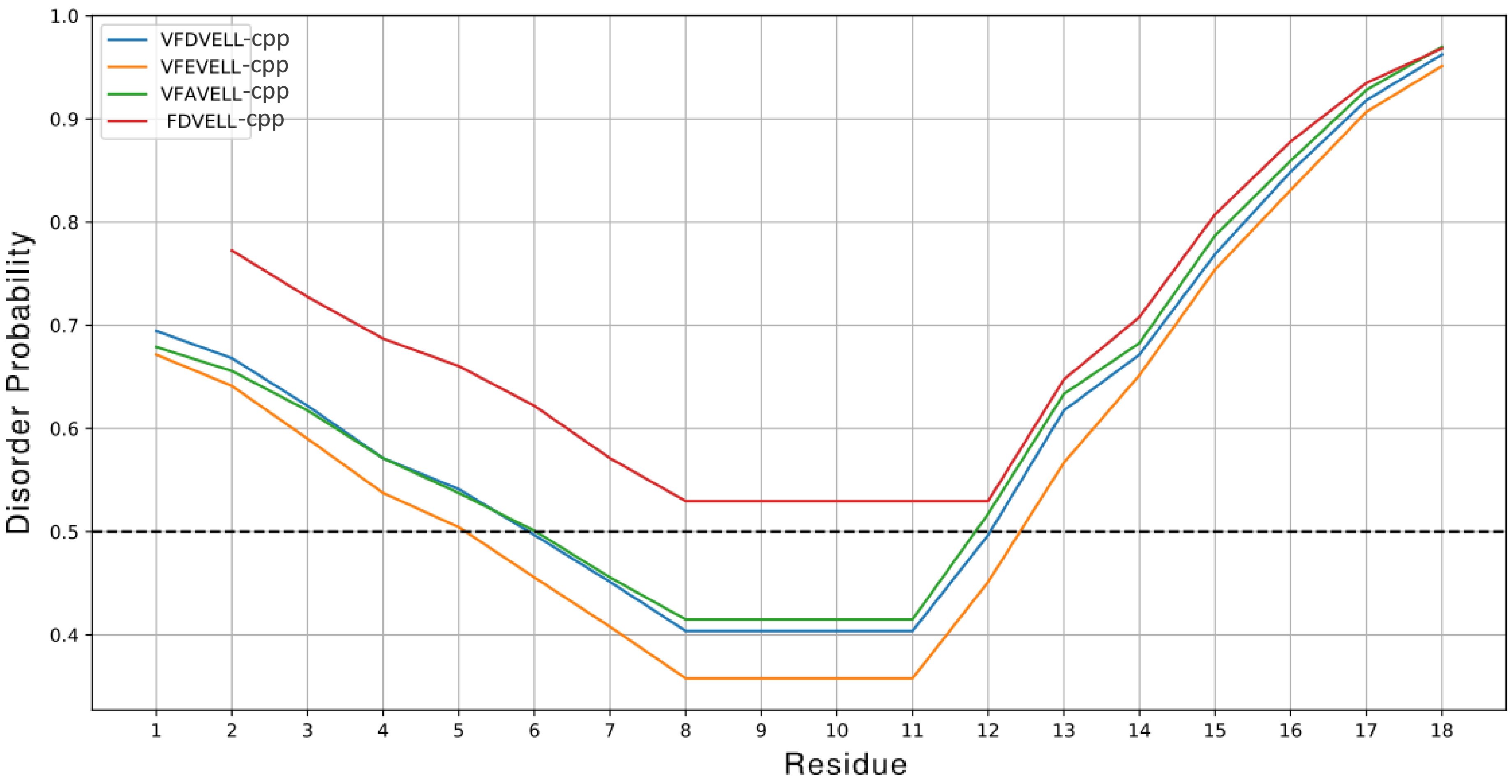

Supplemental Figure S3. Prediction of disordered regions for each peptide, aligned with respect to the last residue, obtained with the IUPred2A server. Relatively long disordered regions are predicted to exist between residues 1 to 5 and 13 to 18, except for the VFD6-TAT peptide, for which the predicted probability of disorder is higher than the decision threshold throughout.

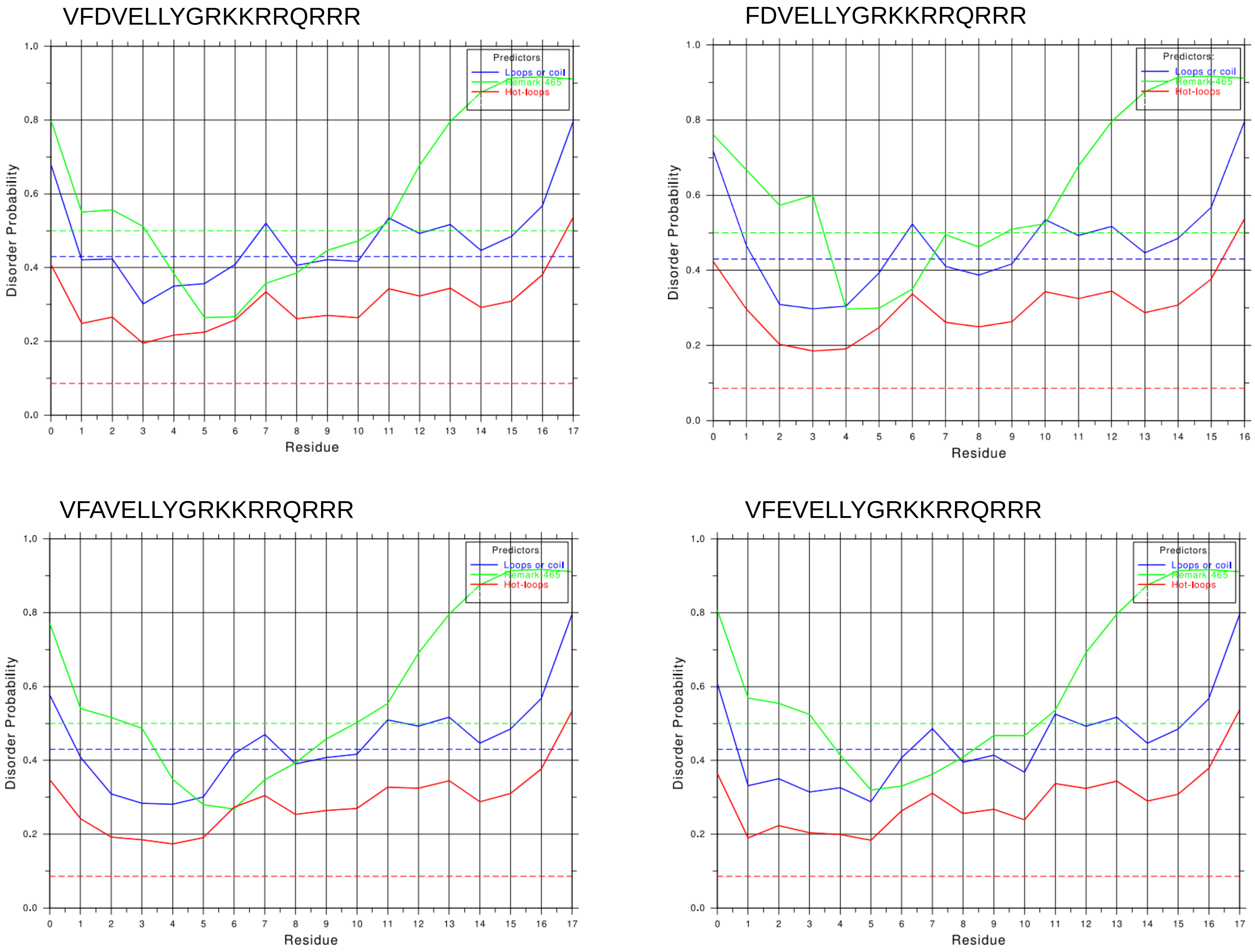

Supplemental Figure S4. Disordered region prediction from the DisEMBL server for indicated peptides. These results were in broad agreement with each other and with the results from the IUPread2A server.

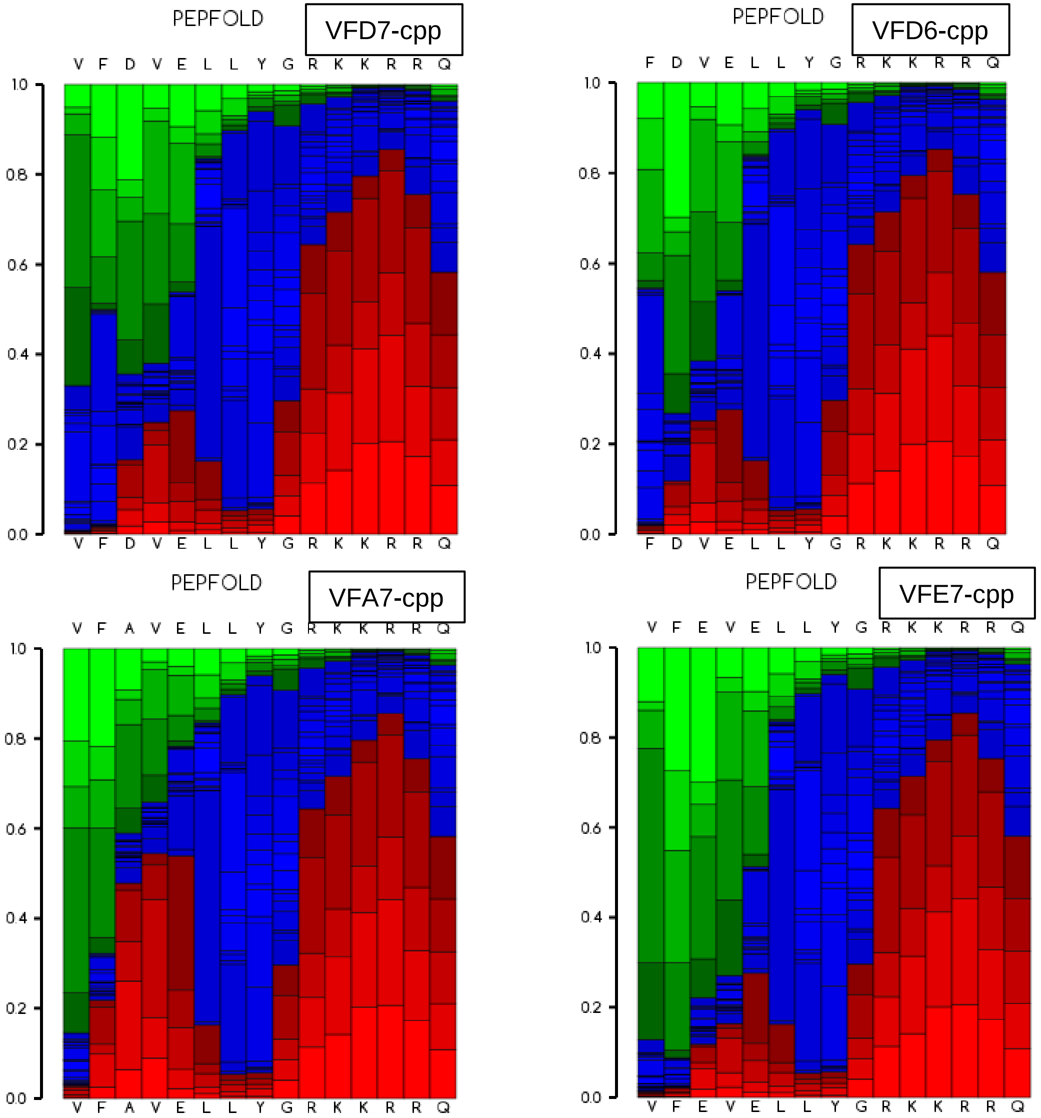

Supplemental Figure S5. Secondary structure prediction results from the PEP-FOLD3 tool for VFD7. The common C-terminal region is predicted to be helical from the first arginine residue onwards with high confidence. The varying N-terminal region shows corresponding greater variability for the first 5 residues, with different degrees of helical or extented propensity, followed by a probable coil conformation for the (L)LYG fragment.

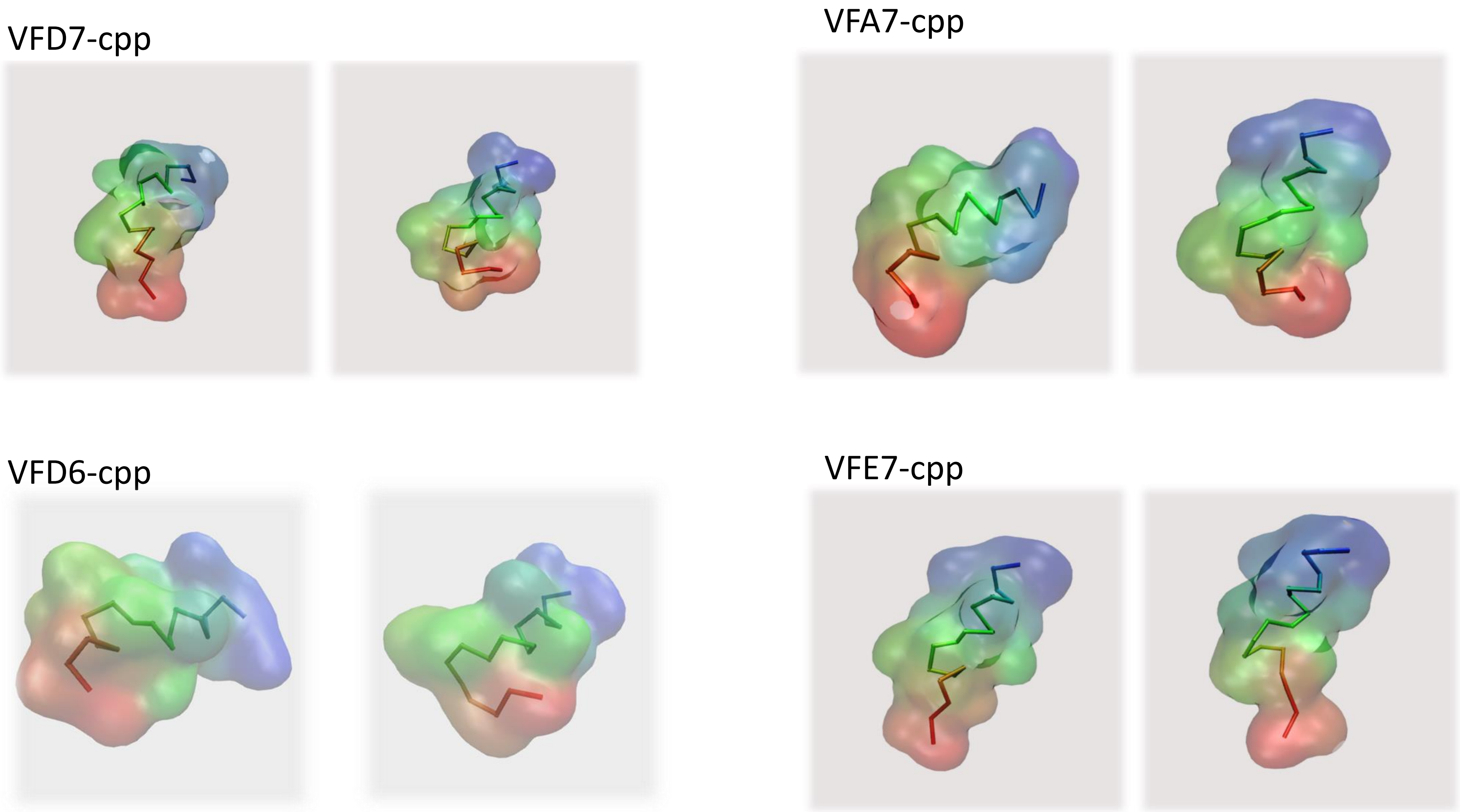

Supplemental Figure S6. Selected models for selected VFD7 derived peptides.

Supplementary chromatograms from peptide synthesis analyses

Quality control chromatograms from peptide synthesis are following below

proteimax

Análise por HPLC

(YGRKKRRQRRR)

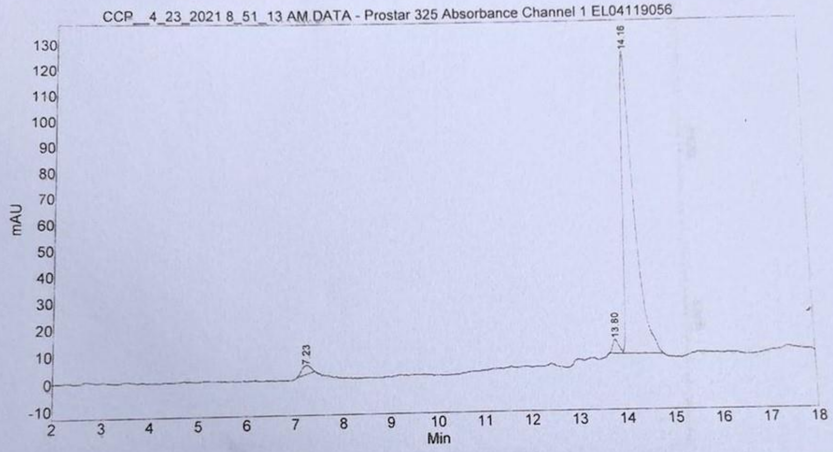

Peak results :

Index	Time [Min]	Area [mAU.Min]	Area % [%]
1	7.23	0.7	2.250
2	13.80	0.8	2.382
3	14.16	30.9	95.367
Total		32.4	100.000

AVE5

Análise por HPLC do peptídeo
(AVELLYGRKKRRQRRR) 16aa

mw = 2085.49

18.50 mg

Add 9mL

[ ] = 2mg/mL

1mh

REPORT

Sample Description:

Structure : AVE5 AR-16
Number : 010250011
Lot No : P190306-TL712396
Column : 4.6×250mm, Kromasil 100-5C18
Solvent A : 0.1% trifluoroacetic in 100% acetonitrile
Solvent B : 0.1% trifluoroacetic in 100% water
Gradient :

	A	B
0.01min	10%	90%
25min	35%	65%
25.1min	100%	0%
30min	STOP	

Flow rate : 1.0 mL/min
Wavelength : 220nm
Volume : 5ul

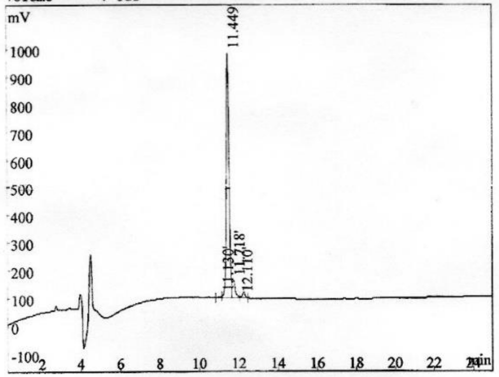

Rank	Time	Conc.	Area	Height
1	11.130	0.2955	24212	5094
2	11.449	90.37	7404638	883068
3	11.718	7.159	586649	62112
4	12.110	2.177	178367	4022
Total		100	8193866	954296

proteimax

EVE5

Análise por HPLC do peptídeo
(EVELLYGRKKRRQRRR) 16aa

mw = 2143.53

18.9 ms

Add 9 mL H_2O

[ ] = 2mg/mL

mL = 1

Structure	: EVE5 ER-16		
Number	: 010250011		
Lot No	: P190306-TL712397		
Column	: 4.6×250mm,Kromasil 100-5C18		
Solvent A	: 0.1% trifluoroacetic in 100% acetonitrile		
Solvent B	: 0.1% trifluoroacetic in 100% water		
Gradient	:	A	B
	0.01min	15%	85%
	25min	40%	60%
	25.1min	100%	0%
	30min	STOP	
Flow rate	: 1.0 mL/min		
Wavelength	: 220nm		
Volume	: 5ul		

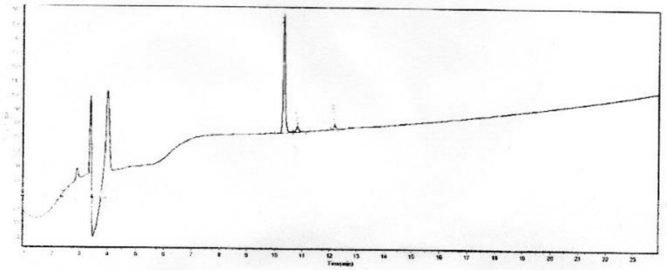

Peak No.	Ret Time	Height	Area	Conc.
1	10.317	81427.609	432595.000	91.4645
2	10.807	3877.303	27491.314	5.8125
3	12.175	2631.559	12878.574	2.7229
Total				100.0000

VFAT7

Análise por HPLC do peptídeo

(VFAVELLYGRKKRRQRRR) 18aa

mw = 2331.80

25mg

Add 12mL H_2O

[ ] = 2 mg/mL

1mM

Sample Description:

Structure:VFAT VR-18

Number: 0200046

Lot No:P190306-TL712395

Column: 4.6mm*250mm, Inertsil ODS-SP

Solvent A: 0.1%Trifluoroacetic in 100% Acetonitrile

Solvent B: 0.1%Trifluoroacetic in 100% Water

Gradient:

	A	B
0.01min	20%	80%
25.00min	45%	55%
25.01min	100%	0%
30.00min	Stop	

Flow rate:1.0ml/min

Wavelength:220nm

Volume:10μl

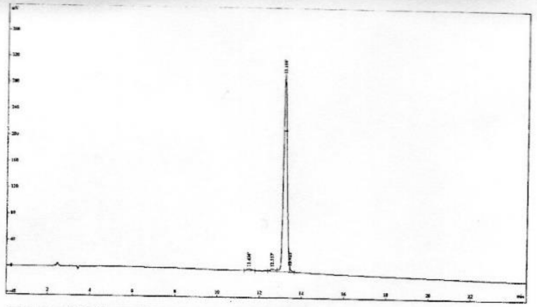

Rank	Time	Conc.	Area	Height
1	11.456	0.9784	37026	2223
2	12.557	1.287	48700	3329
3	13.108	97.29	3681896	303505
4	13.425	0.4395	16632	5323
Total		100	3784254	314380

proteimax

VFE7

Análise por HPLC do peptídeo
(VFEVELLYGRKKRRQRRR) 18 aa

Mw = 2389.84

18.10 mg

Add 7.5 mL H_2O

[ ] = 2.4 mg/mL

1 mM

Product Name:VFE7 VR-18
Instrument No: 0200023
Lot No :P190306-TL712394
Column :4.6*250mm, GS-120-5-C18-BIO
Solvent A :0.1%Trifluoroacetic in 100% Acetonitrile
Solvent B :0.1%Trifluoroacetic in 100% Water

Gradient :

	A	B
0.00min	18%	82%
25min	43%	57%
25.1min	100%	0%
30min	Stop	

Flow rate :1.0ml/min
Wavelength :220nm
Volume :10ul

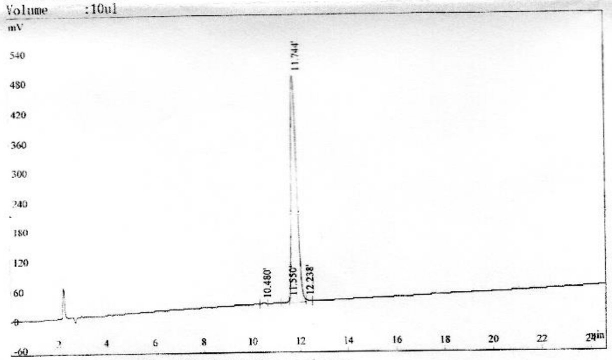

Rank	Time	Conc.	Area	Height
1	10.480	0.2602	18067	2195
2	11.550	0.2008	13940	3371
3	11.744	99.2464	6890442	456113
4	12.238	0.2926	20317	3055
Total		100	6942766	464734

proteimax

Análise por HPLC do peptídeo VFD7-cpp
VFDVELLYGRKKRRQRRR

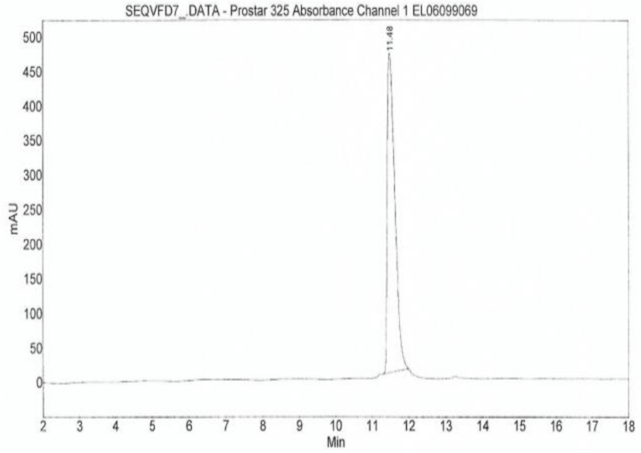

Peak results :

Index	Time [Min]	Area [mAU.Min]	Area % [%]
1	11.48	110.5	100.000
Total		110.5	100.000

Análise por HPLC do peptídeo
(VELLYGRKKRRQRRR)

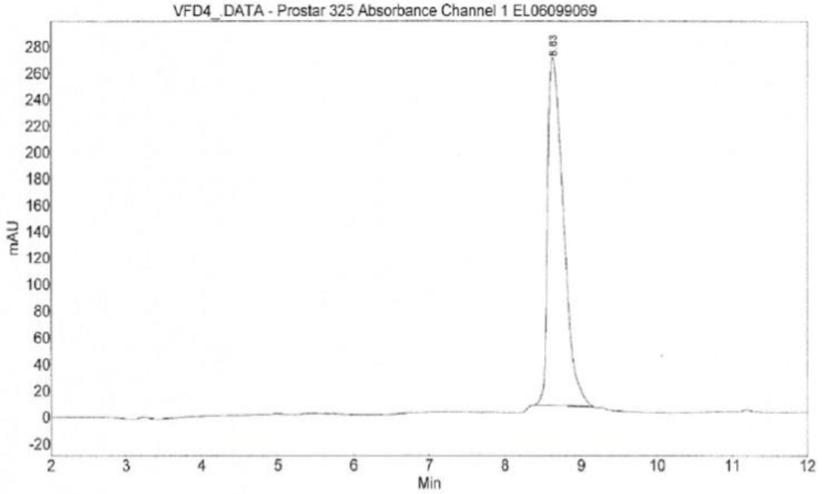

Peak results :

Index	Time [Min]	Area [mAU.Min]	Area % [%]
1	8.63	63.0	100.000
Total		63.0	100.000

Análise por HPLC do peptídeo
(DVELLYGRKKRRQRRR)

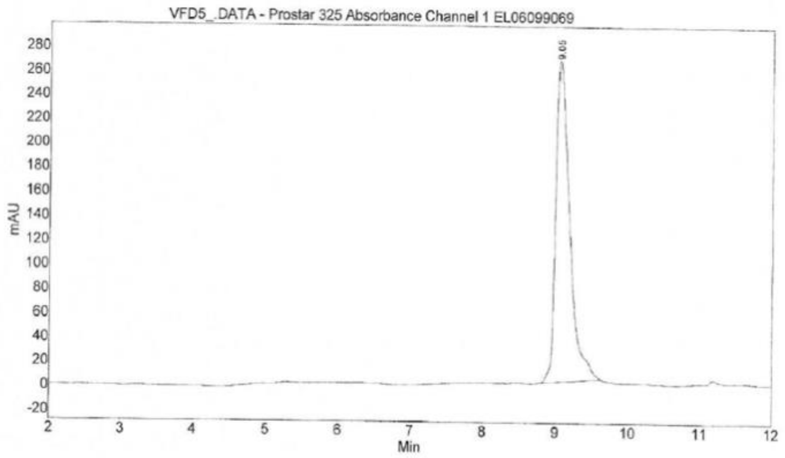

Peak results :

Index	Time [Min]	Area [mAU.Min]	Area % [%]
1	9.05	59.5	100.000
Total		59.5	100.000

Peptide : VFD6

FDVELLYGRKKRRQRRR

System : HPLC 4
Method : Peptide
2 1 Column HPLC 4

Acquired : 8/23/2018 AM

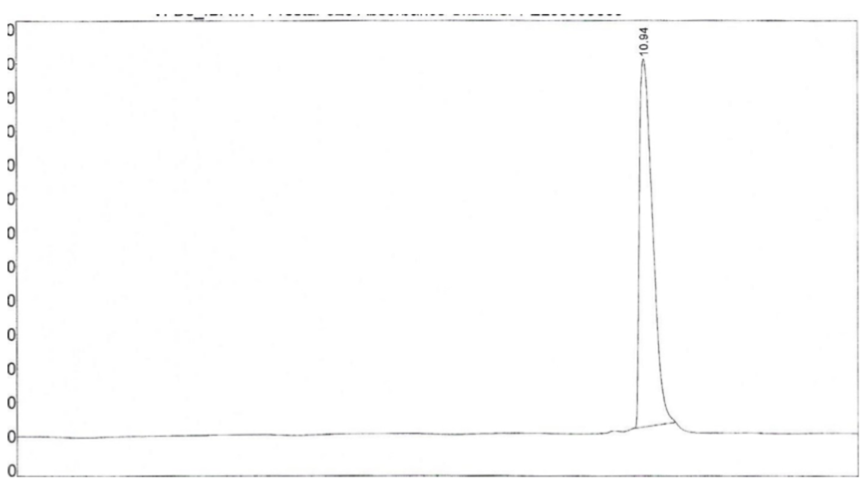

VFD6 DATA - Prostar 325 Absorbance Channel 1

EL06099069 O

2 3 4 5 6 7 8 9 10 11 12 13 14 Min

Peak results :

Index	Time Mi	Area mAUMn	Area % [%]
1	1 .94	1 16.6	98.896
2	16.03	1 .3	1.104
Total		1 17.9	100.000

proteimax

Análise por HPLC do peptídeo
(VFDVELLKLEYGRKKRRQRRR)

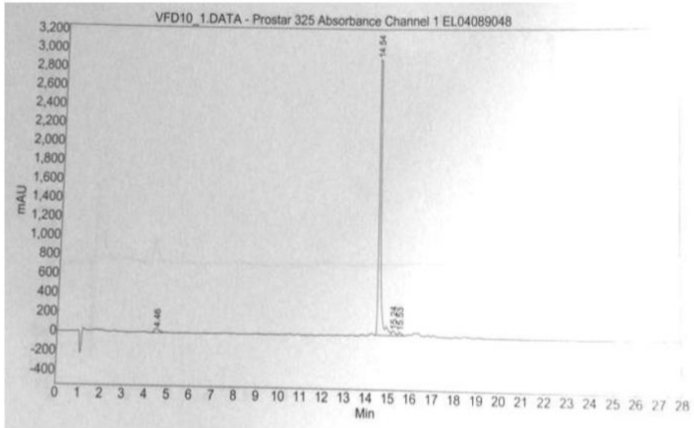

Peak results :

Index	Time [Min]	Area [mAU.Min]	Area % [%]
1	4.46	11.4	2.999
2	14.54	351.5	92.826
3	15.24	9.5	2.501
4	15.53	6.3	1.674
Total		378.7	100.000
